# Fully covered metallic stents for anastomotic biliary strictures after living donor liver transplantation

**DOI:** 10.1002/deo2.225

**Published:** 2023-03-27

**Authors:** Naohiro Komatsu, Eisuke Ozawa, Masanori Fukushima, Hironori Sawase, Kazuyoshi Nagata, Satoshi Miuma, Hisamitsu Miyaaki, Akihiko Soyama, Masaaki Hidaka, Susumu Eguchi, Kazuhiko Nakao

**Affiliations:** ^1^ Department of Gastroenterology and Hepatology Nagasaki University Graduate School of Biomedical Sciences Nagasaki Japan; ^2^ Department of Surgery Nagasaki University Graduate School of Biomedical Sciences Nagasaki Japan

**Keywords:** anastomotic biliary stricture, fully covered self‐expandable metallic stent, living donor liver transplantation, plastic stent, cholangitis

## Abstract

**Objectives:**

Anastomotic biliary strictures (ABSs) are common complications following living donor liver transplantation (LDLT). We evaluated the feasibility of a novel removable, intraductal, fully covered, self‐expandable metallic stent (FCSEMS) for the treatment of ABSs following LDLT.

**Methods:**

Nine patients with duct‐to‐duct ABSs that developed following LDLT were prospectively enrolled in this study. We placed a short FCSEMS with a long lasso and middle waist formation in each patient's ABS above the papilla and removed it 16 weeks later.

**Results:**

The FCSEMS placements were successful in all nine cases. Four patients experienced mild cholangitis, which was resolved with conservative treatment. Additionally, there was one case of distal migration. The FCSEMSs were successfully removed from all the patients, and the clinical success rate was 100%. Stricture recurrence occurred in one (11.1%) patient during the follow‐up period.

**Limitations:**

The small number and lack of comparison with other types of FCSEMSs and plastic stents.

**Conclusions:**

Intraductal placement of FCSEMSs is useful for treating refractory ABSs after LDLT, although further studies are required with larger sample sizes.

## INTRODUCTION

Liver transplantation (LT) is effective for treating patients with end‐stage liver disease.^1^, ^2^ However, various complications can occur. Biliary complications are especially common after LT,^3^ and their incidence is 5%–32%.^4^, ^5^, ^6^ Biliary strictures post‐LT are usually anastomotic, but non‐anastomotic strictures include relapse of primary sclerosing cholangitis. The incidence of anastomotic biliary strictures (ABSs) is 6%–12% after orthotopic liver transplantation^7^, ^8^ and 16%–32% after living donor liver transplantation (LDLT).^9^, ^10^

ABSs commonly occur as late complications, approximately 5–8 months after transplantation.^6^ Therefore, it is necessary to perform imaging, such as abdominal ultrasonography, computed tomography, and magnetic resonance cholangiopancreatography, at an early stage when hepatobiliary enzymes rise to determine whether ABSs are present. If biliary strictures are strongly suspected, endoscopic retrograde cholangiopancreatography (ERCP), which can be used for both diagnosis and treatment, should be performed.

The use of plastic stents (PSs) and balloon dilation is common for treating ABSs that develop after LDLT,^11^ and success rates with these methods range from 37%–96%.^9^, ^10^, ^12^, ^13^, ^14^, ^15^, ^16^ However, balloon dilation and PSs are usually replaced at 3–6 month intervals, thereby requiring frequent endoscopic procedures. Furthermore, there is a strong possibility of stricture recurrence.

Self‐expandable metallic stents (SEMSs) are useful for treating ABSs after LT because of their wider biliary dilatation capability. Compared to PSs, SEMSs prolong the duration of bile duct patency and reduce the need for reintervention. Additionally, ABS treatment success rates with metallic stents range from 75% to 100%,^17^, ^18^, ^19^ and the recurrence rates are low (15%–24%).^17^, ^18^

In this study, we aimed to evaluate the long‐term efficacy and safety of non‐flared, fully covered, self‐expandable metallic stents (FCSEMSs) for the treatment of ABSs after LDLT that are not resolved with conventional endoscopic treatments using plastic stents and balloon dilatation.

## METHODS

### Study design and patient selection

**FIGURE 1:**
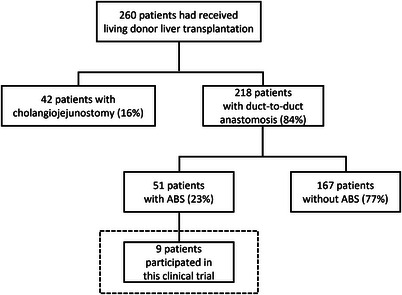
Flowchart of patient inclusion. ABS, anastomotic biliary strictures.

This was a single‐center, prospective, non‐randomized pilot study performed from July 2019 to May 2021. Figure 1 depicts a flowchart of the patient inclusion process. From August 1997 onward, 260 patients received LDLT in our hospital. Two hundred eighteen patients had duct‐to‐duct anastomosis, of which 51 patients had clinical symptoms or elevated hepatobiliary enzyme levels indicating bile duct obstruction; those with an ABS after LDLT proven by imaging (e.g., ultrasonography, computed tomography, or magnetic resonance cholangiopancreatography), and those with persistent biliary strictures after endoscopic treatment using PS, with or without balloon dilation. Nine patients participated in this clinical trial. The exclusion criteria were as follows: inability to tolerate endoscopic treatment, suspected malignant biliary stricture, biliary stricture due to a benign tumor, surgically altered gastrointestinal anatomy, the severe bleeding tendency (i.e., platelet count <50,000/mm^3^, prothrombin time international normalized ratio >1.5, or taking antithrombotic drugs), and those who refused to participate. Written informed consent for the endoscopic procedure was obtained from all the patients. Immunosuppression therapy with tacrolimus or cyclosporin and steroids was initiated after the LDLT procedure to avoid and treat graft rejection, and basiliximab or mycophenolate mofetil was administered to patients with renal dysfunction.

This study was approved by the Institutional Review Board of our hospital, and it was registered in the UMIN Clinical Trial Registry (UMIN000036910).

### Endoscopic procedure and follow‐up

**FIGURE 2:**
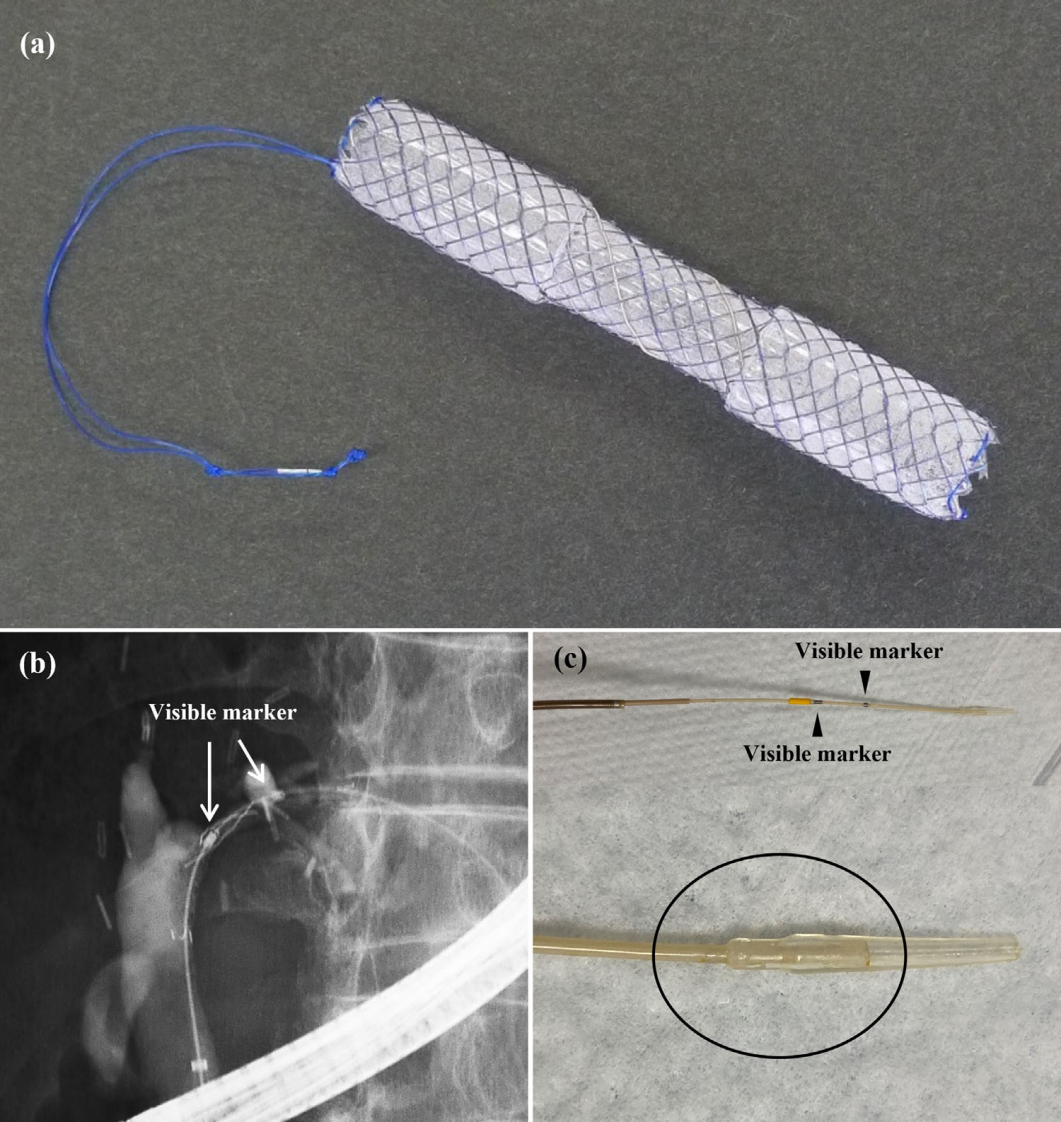
(a) Intraductal fully covered self‐expandable metallic stent (BONASTENT M‐Intraductal; Standard Sci Tech Inc., Seoul, South Korea). This fully covered self‐expandable metallic stent has a slightly constricted central part to prevent migration and a long lasso for easy removal. There is a visible crosswire in the center for excellent visibility. (b, c) The tip of the inner cylinder and the visible marker are configured as shown in the figure.

Before participating in this study, each patient had a plastic stent placed at least once, and endoscopic sphincterotomy was not performed after the LDLT procedure. The intra‐ductal FCSEMS (BONASTENT M‐Intraductal; Standard Sci Tech Inc., Seoul, South Korea) we used has a slightly constricted central portion to prevent migration and dislocation (Figure 2).^20^ We performed the ERCP using a side‐viewing duodenoscope (TJF‐260V or JF‐260V; Olympus Medical, Tokyo, Japan) under moderate sedation with intravenous administration of midazolam and pethidine hydrochloride. Scopolamine butylbromide or glucagon was used as a gastroduodenal antispasmodic agent. Antibiotic administration was routinely initiated immediately before the ERCP procedure. Non‐invasive blood pressure measurements, pulse oximetry, and electrocardiography were used to continuously monitor vital signs during the procedure.

**FIGURE 3:**
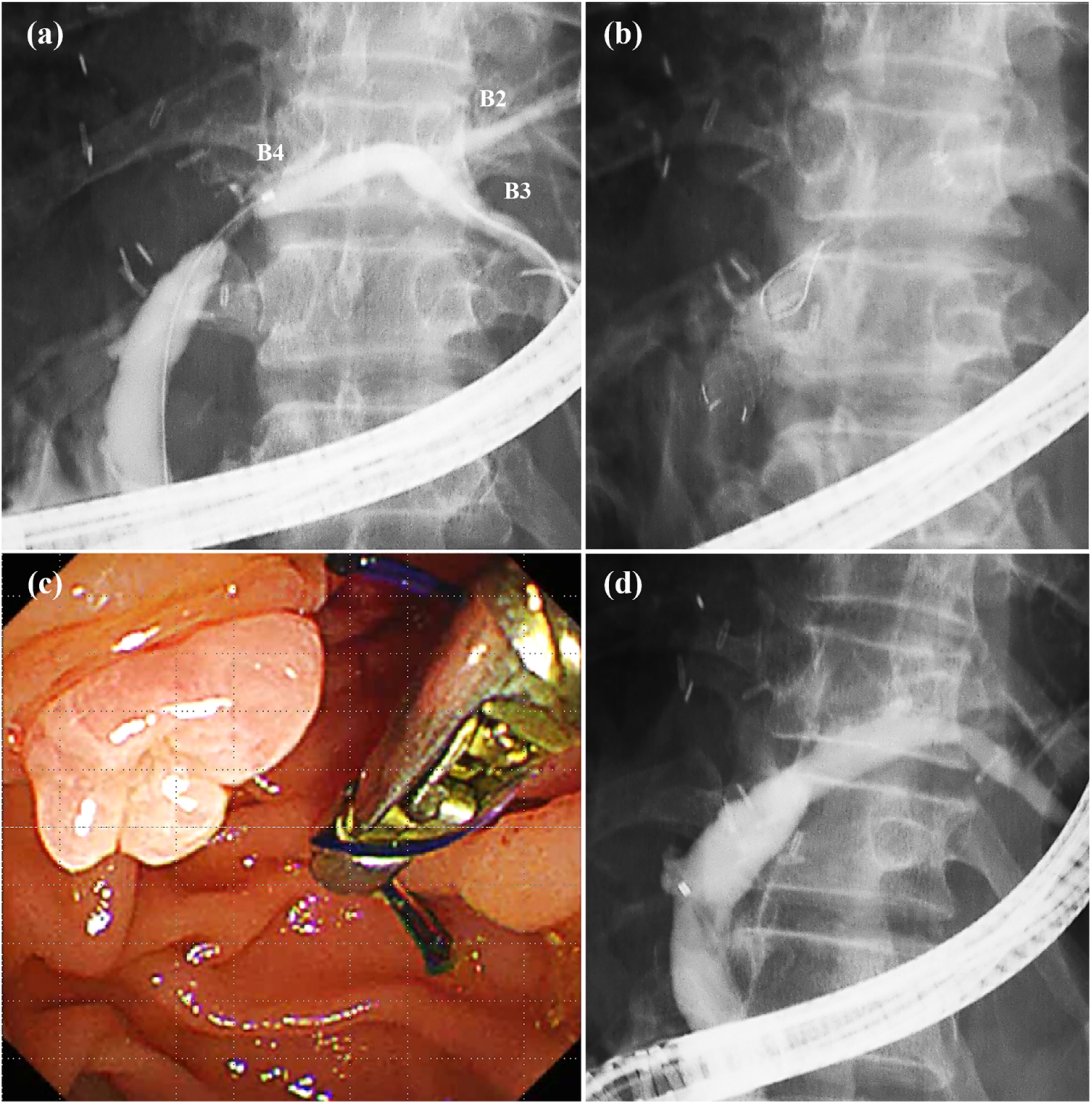
This patient was a 55‐year‐old male with an anastomotic biliary stricture that developed after left lobe living donor liver transplantation. (a) Cholangiography before fully covered self‐expandable metallic stent (FCSEMS) placement. Since the confluence of B2 and B3 was far from the anastomotic site, it was believed that there was no risk of obstructing the biliary branch with the FCSEMS. A guidewire was placed in B3 through the left hepatic duct. (b) An FCSEMS was placed in the left hepatic duct. The opaque FCSEMS crosswire was aligned with the center of the anastomotic stricture. The length of the FCSEMS used, in this case, was 40 mm. (c) The FCSEMS was removed successfully after 16 weeks, without resistance, using rotatable forceps. (d) Cholangiography after FCSEMS removal. Anastomotic biliary stricture resolution was achieved 16 weeks after stenting. Recurrence of the stricture has not been observed for more than 15 months.

A hydrophilic guidewire (VisiGlide2; Olympus Medical or Revowave UltraHard; Piolax Medical, Kanagawa, Japan) was placed through the ABS and into the hepatic bile duct (Figure 3a). Cholangiography was performed to determine whether the catheter could pass through the ABS without resistance and to evaluate the confluence of the branches. If there was resistance during catheter insertion, the stricture was dilated with a 6‐ or 8‐mm dilation balloon (REN; Kaneka Medix Corp., Osaka, Japan). When placing each FCSEMS, it was important to align the crosswire with the center of the stricture (Figure 3b), and it was critical to select an FCSEMS length that did not obstruct the biliary branches. If obstruction was unavoidable, we placed a PS side‐by‐side as a rescue stent.^21^ After 16 weeks, each FCSEMS was removed through the working channel of the duodenoscope by grasping the lasso with forceps (Figure 3c). Cholangiography was performed to confirm that the ABS was resolved (Figure 3d).

**FIGURE 4:**
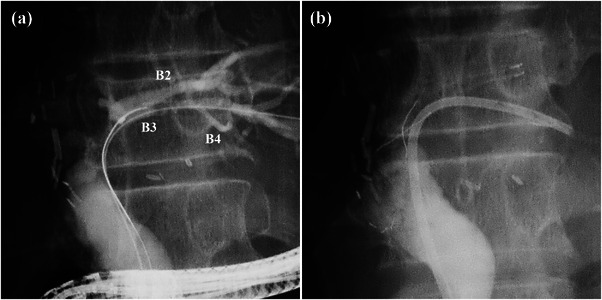
This patient was a 61‐year‐old male with an anastomotic biliary stricture that developed after left lobe living donor liver transplantation. (a) Cholangiography before fully covered self‐expandable metallic stent (FCSEMS) placement. The confluence of B2 and B3 was close to the anastomotic site and B4 merged with B3. Therefore, it was highly likely that the FCSEMS occluded one of the biliary branches. (b) After a plastic stent (PS) was placed in B3 so as not to obstruct B4, an FCSEMS was placed in B2. Both the FCSEMS and PS were placed intraductally.

In cases of duct‐to‐duct anastomosis, the biliary branch differs depending on which lobe is grafted. For a left lobe graft, the FCSEMS is often placed in B2 or B3, but this creates a risk of obstructing the biliary branch (Figure 4a). Therefore, it may be necessary to place a PS in the biliary branch, away from the FCSEMS (Figure 4b).

**FIGURE 5:**
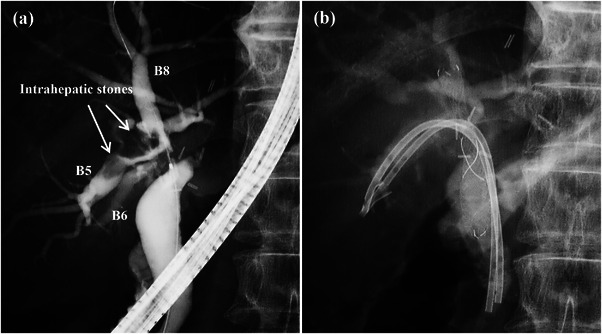
This patient was a 73‐year‐old male with an anastomotic biliary stricture that developed after right‐lobe living donor liver transplantation. (a) Cholangiography before fully covered self‐expandable metallic stent (FCSEMS) placement. Since the confluence of the anterior and posterior segment branches was close to the anastomotic site, it was highly likely that the FCSEMS occluded the other biliary branch. In addition, large intrahepatic stones were present in the anterior segment branch (arrow), and it was decided that an FCSEMS should be placed in B8 after placing plastic stents in B5 and B6. (b) An FCSEMS was placed in B8, and plastic stents were placed in B5 and B6 to prevent obstruction of the biliary branches. The FCSEMS and plastic stents were placed intraductally.

For a right lobe graft, the upper end of the FCSEMS may span the confluence of the anterior and posterior segment branches (Figure 5a). If the FCSEMS is placed in the anterior segment branch, the PS may have to be retained on the posterior segment branch (Figure 5b).

We followed the patients on an outpatient basis with clinical examinations and blood tests, including hepatobiliary enzymes, at 1, 3, 6, 9, and 12 months after removal. Abdominal imaging (computed tomography or magnetic resonance cholangiopancreatography) was used to investigate signs of recurrence, and ERCP was performed if recurrence was strongly suspected and biliary drainage was required.

### Definition of events

Technical success was defined as the successful positioning of the FCSEMS along the stricture with satisfactory self‐expansion. Clinical success was defined as the resolution of the stricture and clinical symptoms, including jaundice, after FCSEMS removal. Stricture resolution was defined as visualization of the donor's bile duct from contrast injected into the recipient's bile duct and as an extension of 3 mm or more of the stricture. Adverse events (AEs) after placement were defined and recorded according to the American Society for Gastrointestinal Endoscopy guidelines. Early AEs were defined as those occurring less than 30 days after placement, and late AEs were those occurring 30 days or later after placement. Stricture recurrence was defined as a stricture demonstrated by cholangiography after initial clinical success.

### Primary and secondary outcomes

The primary outcome of this study was the clinical success rate (stricture resolution and clinical symptoms). The secondary outcomes were the technical success rate, stricture recurrence, early AEs (<30 days after placement), and late AEs (≥30 days after placement).

### Statistical analysis

The patient demographic and clinical characteristics are presented as the median (range) or median (interquartile range) for continuous variables and frequency (percentage) for categorical variables. The statistical analyses were performed using SPSS version 23.0 (IBM Corp., Armonk, NY, USA).

## RESULTS

Nine patients were enrolled in this study and underwent FCSEMS placement. The patients’ baseline characteristics are described in Table 1. The median patient age was 63.0 years (range, 49–73 years), and six patients were men (66.7%). The most common indications for LDLT were viral liver cirrhosis (seven patients, 77.8%), nonalcoholic steatohepatitis (one patient, 11.1 %), and primary biliary cholangitis (one patient, 11.1%). Seven patients had hepatocellular carcinoma before the LDLT. All patients received transplants from living donors with duct‐to‐duct anastomosis. One patient (11.1%) underwent right lobe transplantation, and the remaining eight (88.9%) underwent left lobe transplantation. The median time to ABS onset was 5 months (range, 3–32 months). Five patients developed common bile duct stones, and three had intrahepatic stones.

**TABLE 1:** Baseline characteristics of the patients.

Characteristics	All patients (*N* = 9)
Age (years), median (range)	63 (49–73)
Sex, *n* (%)	
Male	6 (66.7)
Female	3 (33.3)
Indication for LDLT, *n* (%)	
Viral liver cirrhosis	7 (77.8)
Nonalcoholic steatohepatitis	1 (11.1)
Primary biliary cholangitis	1 (11.1)
Hepatocellular carcinoma, *n* (%)	5 (55.6)
Liver graft, *n* (%)	
Right lobe	1 (11.1)
Left lobe	8 (88.9)
Time to ABS onset (months), median (range)	5 (3–32)
Bile duct stone, *n* (%)	
Common bile duct stone	5 (55.6)
Intrahepatic stone	3 (33.3)

Abbreviations: ABS, anastomotic biliary stricture; LDLT, living donor liver transplant.

The treatment results are summarized in Table 2. The FCSEMSs were successfully placed along the strictures for all the patients, and the technical success rate was 100%. The median procedure time was 42 min (range, 31–102 min), and a 10 mm diameter FCSEMS was used for all the patients. The length of the FCSEMS used was 4 cm in three (33.3%) patients, 5 cm in four (44.4%) patients, and 6 cm in two (22.2%) patients. Pre‐dilation was defined as balloon dilation before placement of the FCSEMS, and four patients (44.4%) underwent pre‐dilation. PSs were placed side‐by‐side as a rescue stent in four cases (44.4%) because of the risk of bile duct branch obstruction. Cholangitis occurred in four patients (44.4%) as an early AE; however, all the cases were mild and resolved with conservative antibiotic treatment. No pancreatitis was observed. Distal migration of the FCSEMS occurred in one case, although it remained in the bile duct and stricture resolution occurred without re‐intervention. Proximal migration of the FCSEMS or FCSEMS‐induced strictures was not observed.

**TABLE 2:** Summary of the treatment results.

Characteristics	All patients (*N* = 9)
Technical success, *n* (%)	9 (100)
Procedure time (minutes), median (range)	42 (31–102)
Diameter of FCSEMS (mm), *n* (%)	
10	9 (100)
Length of FCSEMS (cm), *n* (%)	
4/5/6	3 (33.3)/4 (44.4)/2 (22.2)
Pre‐dilation, *n* (%)^†^	4 (44.4)
Plastic stent placement, *n* (%)	4 (44.4)
Early adverse events, *n* (%)	
Cholangitis^‡^	4 (44.4)
Pancreatitis	0 (0)
Late adverse events, *n* (%)	
Cholangitis	2 (22.2)
FCSEMS distal migration	1 (11.1)
FCSEMS‐induced stricture, *n* (%)	0 (0)

Abbreviation: FCSEMS, fully covered self‐expandable metallic stent.

^†^ Pre‐dilation was defined as balloon dilation before FCSEMS placement.

^‡^ All patients with cholangitis were mild and resolved with conservative treatment.

The outcomes of interest are shown in Table 3. One FCSEMS was removed at 6 weeks due to segmental cholangitis, while the others were removed at 16 weeks, as planned. The median stenting duration was 16 weeks (range, 6–16 weeks). In all cases, the FCSEMS was removed successfully with rat‐tooth forceps and the clinical success rate was 100%. The patient who had the stent removed at 6 weeks experienced stricture resolution without re‐intervention. The median anastomotic diameter after FCSEMS removal was 6.3 mm (range, 3.5–7.1 mm). During the median follow‐up of 688 days (range, 562–842 days) after FCSEMS removal, stricture recurrence occurred for one (11.1%) patient after clinically successful resolution.

**TABLE 3:** Outcomes of interest.

Characteristics	All patients (*N* = 9)
Duration of stenting (weeks), median (range)^†^	16 (6–16)
Success of FCSEMS removal, *n* (%)	9 (100)
Clinical success, *n* (%)	9 (100)
Anastomotic diameter after FCSEMS removal (mm), median (range)	6.3 (3.5–7.1)
Duration of follow‐up (days), median (range)	688 (562–842)
Recurrence of the stricture, *n* (%)^‡^	1 (11.1)
Duration till recurrence (days)	288

^†^ One FCSEMS was removed at 6 weeks due to segmental cholangitis.

^‡^ The recurrent case was a patient who had to have the stent removed at 6 weeks, and FCSEMS was placed again.

The case details are presented in Table 4. ERCP was performed ≥10 times for more than half of the patients before FCSEMS placement. PS placement and pre‐dilation for the patients were as shown. We successfully removed common bile duct stones from five patients. Two of the three patients with intrahepatic stones required electrohydraulic lithotripsy due to large stones. Complete stone clearance was achieved at the time of the FCSEMS removal. All the strictures were resolved, however, the patient who had the FCSEMS removed after 6 weeks experienced recurrence. We repeated the FCSEMS procedure for this patient and removed it 16 weeks later, as with the other patients, and clinical success was achieved.

**TABLE 4:** Case details.

No.	Liver graft	No. of ERCP before FCSEMS placement	Duration of FCSEMS stenting (weeks)	PS placement/ Pre‐dilation	Common bile duct	Intrahepatic duct	Stone removal	EHL	Resolution of the stricture	Recurrence of the stricture
1	Left lobe	2	6	Yes / No	No	No	−	−	Yes	Yes
2	Left lobe	14	16	No / No	Yes	Yes	Yes	Yes	Yes	No
3	Left lobe	3	16	No / No	No	No	−	−	Yes	No
4	Left lobe	3	16	No / No	No	No	−	−	Yes	No
5	Left lobe	1	16	No / No	No	No	−	−	Yes	No
6	Left lobe	19	16	Yes / Yes	Yes	No	Yes	No	Yes	No
7	Left lobe	11	16	No / Yes	Yes	No	Yes	No	Yes	No
8	Left lobe	11	16	Yes / Yes	Yes	Yes	Yes	No	Yes	No
9	Right lobe	17	16	Yes / Yes	Yes	Yes	Yes	Yes	Yes	No

Abbreviations: EHL, electrohydraulic lithotripsy; ERCP, endoscopic retrograde cholangiopancreatography; FCSEMS, fully covered self‐expanding metallic stent; PS, plastic stent.

## DISCUSSION

ABS is common after liver transplantation, more so for LDLT than for orthotopic liver transplantation.^22^ This is because there is a diameter mismatch between the donor hepatic duct and the recipient common bile duct for LDLT recipients. PS placement and balloon dilation are commonly performed to treat ABSs after LDLT,^11^ but the success rates with these methods range from 37% to 96%.^9^, ^12^, ^13^, ^14^, ^15^, ^16^ Additionally, multiple and long‐term treatments are required before the stricture is resolved.^23^, ^24^ To address these problems, the temporary placement of FCSEMSs for ABSs after LDLT has increased recently.^18^, ^25^, ^26^

Some reports suggest that FCSEMSs have better stricture resolution and recurrence rates than PSs,^19^, ^27^, ^28^ as large‐diameter FCSEMSs can induce stricture resolution in a single session. The FCSEMS patency period is longer than that of PSs and is more cost‐effective because of the reduced number of ERCP procedures required.^28^ However, FCSEMS placement is more complex than PS placement because the metallic stent can obstruct the bile duct side branches.

A consensus has not been reached about the duration of the FCSEMS placement as reports indicate a range from 2 to 4 months^18^, ^25^, ^26^, ^29^; we chose a period of 16 weeks. In a study examining the FCSEMS placement period,^30^ the median period was 119 days (93–161 days) for the group that achieved stricture resolution and 68 days (57.5–80 days) for the group that did not. In our study, it took 58 days to resolve the strictures in 50% of the patients and approximately 120 days for 80% of the patients to achieve resolution. This may have been because a long‐term duration more readily results in stricture resolution.^30^

In the current study, only one patient developed segmental cholangitis, had the FCSEMS removed after 6 weeks, and experienced a recurrence 10 months after resolution. Another FCSEMS was placed, with a PS placed side‐by‐side, and remained for 16 weeks without the patient developing cholangitis. The stricture resolved again, indicating that FCSEMSs can be successfully placed repeatedly, much like PSs.

Previous reports using FCSEMS for ABS after liver transplantation are summarized in a table for each intraductal FCSEMS (ID‐FCSEMS) and non‐ID‐FCSEMS (Table 5).^18^, ^23^, ^25^, ^26^, ^31^, ^32^, ^33^, ^34^ Both have a high stricture resolution rate and low recurrence rate of the stricture. However, it seems that ID‐FCSEMS is associated with fewer incidents, especially pancreatitis.

**TABLE 5:** Summary of outcomes in studies using a metallic stent.

	Author	No. of ABS	Type of metallic stent	Mean stent duration (months)	Mean follow‐up (months)	Resolution of the stricture, number (%)	Recurrence of the stricture, number (%)	Adverse events ^†^ (%)
ID‐FCSEMS	Hu et al., 2011	13	FCSEMS (a new stent braided with nitinol wire and covered with silicone membrane)	5.4 ± 1.7	12.1 ± 8	12/13 (92.3)	1/12 (8.3)	1/13 (7.7) Pancreatitis 1 (mild)
	Moon et al., 2012	21	FCSEMS (BONASTENT M‐Intraductal; Standard Sci Tech Inc.)	4.2	13.8	20/21 (95.2)	0/20 (0)	0/21 (0)
3	Aepli et al., 2017	31	FCSEMS (a new stent braided with characteristic waist at mid‐portion and string)	3.8	12.8	29/29 (100)	7/29 (24.1)	1/31 (3.2)
	Yoo et al., 2020	32	FCSEMS (BONASTENT M‐Intraductal; Standard Sci Tech Inc.)	3.4	21.3	26/32 (81.3)	3/26 (11.5)	5/32 (15.6) Pancreatitis 0
	Current study	9	FCSEMS (BONASTENT M‐Intraductal; Standard Sci Tech Inc.)	4	22.9	9/9 (100)	1/9 (11.1)	4/9 (44.4) Pancreatitis 0
Non‐ID‐FCSEMS	Chaput et al., 2010	22	PCSEMS (Wallstent partially covered with Permalume; Boston Scientific)	2	12 ± 1.9	19/22 (86.4)	9/19 (47.3)	5/22 (22.7) Pancreatitis 0
	Tarantino et al., 2012	39	FCSEMS (Niti‐S ComVi, Taewoong Medical)	2	22.1 ± 10	28/39 (71.8)	4/28 (14.3)	Unknown
	Tarantino et al., 2012	15	FCSEMS (Niti‐S ComVi, Taewoong Medical)	2	14.4 ± 2.2	8/15 (53.3)	2/8 (25.0)	Unknown
	Jimenez‐Perez et al., 2016	41	FCSEMS (Wallflex, Boston Scientific)	4.5 ± 2.2 (no recurrence) 3.8 ± 1.2 (recurrence)	27.8 ± 18.3 (no recurrence) 29.5 ± 3.2 (recurrence)	41/41 (100)	9/41 (22.0)	13/41 (31.7) Pancreatitis 8 (mild 5, severe 3)
	Martins et al, 2018	30	FCSEMS (Wallflex, Boston Scientific)	4.6± 2.2	35.9 ± 24.2	25/30 (83.3)	8/25 (32.0)	14/60 (23.3) Pancreatitis 8 (mild 2, moderate 5, severe 1)

Abbreviations: ID‐FCSEMS, intraductal fully covered self‐expandable metallic stent; PCSEMS, partially covered self‐expandable metallic stent.

^†^ Pancreatitis, cholecystitis, cholangitis, sepsis, and abdominal pain, except for stent migration.

We considered using an indwelling PS for B4; however, we chose not to because even if a guidewire can be inserted into B4, it is often difficult to place a PS there due to the sharp bending where B4 branches. Additionally, we believed that segmental cholangitis would not occur if the edge of the FCSEMS was placed so as not to span the B4 confluence. Since the central part of the FCSEMS is constricted, we speculated that the B4 branches would not be completely obstructed. Alternatively, we placed a PS in B3 and an FCSEMS in B2 for the patient in which B4 joined B3.

**FIGURE 6:**
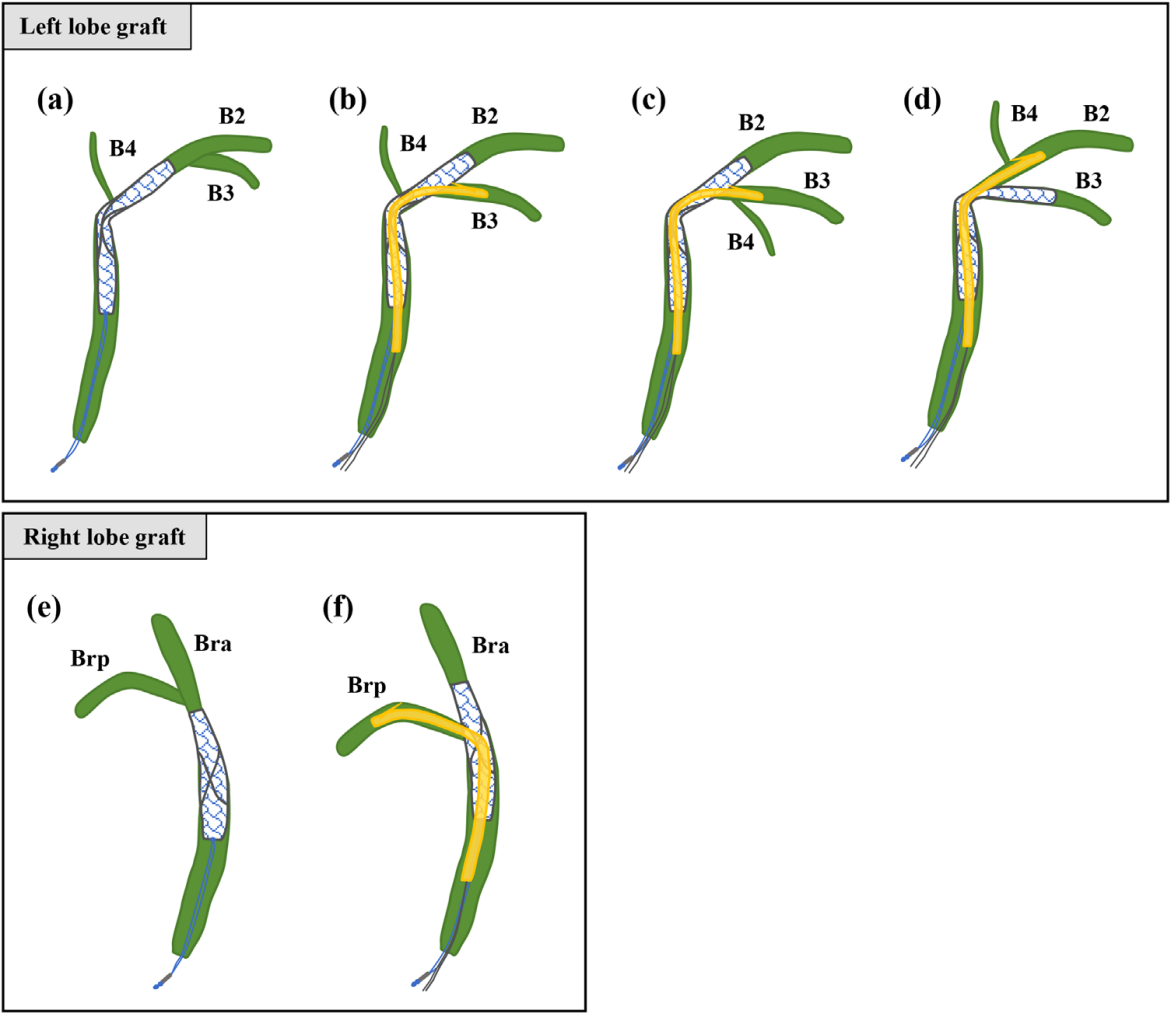
A diagram of fully covered self‐expandable metallic stent (FCSEMS) and plastic stent (PS) placement. (a, b) A case where B4 merges near the anastomotic site. It is technically difficult to place a PS in B4, so it is preferable to place an FCSEMS in B2 so that B4 overlaps the waist of the FCSEMS. (c, d) A case where B4 merges with B2 or B3. The FCSEMS should be placed in the biliary branch in which B4 has not merged. Otherwise, there is a risk that the end of the FCSEMS will overlap B4 and cause segmental cholangitis. (e, f) If the anastomotic site and confluence of the anterior (Bra) and posterior (Brp) segment branches are separated, an FCSEMS alone is enough, but if it is close to the other branch, we recommend placing an FCSEMS in the anterior segment branch and a PS in the posterior segment branch

Figure 6 shows a summary of the methods that can be used to place FCSEMSs and PSs for the treatment of ABSs after LDLT for various bile duct confluences.

While FCSEMSs are particularly useful for treating ABSs, the immediate expanding force after release is not strong. A 10 mm diameter stent was used in all the cases in this study; however, the intrahepatic bile duct was narrow in one patient, and an 8 mm diameter stent was attempted first. When the inner cylinder was removed, the stent got caught and became deviated, so a 10 mm diameter stent was ultimately used. The hook on the tip of the inner cylinder might have gotten caught in the poorly expanded part of the stent or the ABS (Figure 2c). Therefore, we performed a pre‐dilation on four patients in whom there was resistance when the catheter was inserted. This allowed for easy insertion and smooth removal of the inner cylinder. We suggest that when using a PS together with an FCSEMS, pre‐dilation should be performed because PSs interfere with FCSEMSs.

Previously it was thought that early complications, such as cholangitis, were slightly more prevalent with FCSEMSs than with PSs, but recent reports have shown that there is no significant difference.^28^ The high rate of cholangitis in our study may have been the result of the patients taking immunosuppressants, as they might promote cholangitis development.

Jang et al.^28^ found that there was no significant difference in the incidences of pancreatitis among patients that underwent FCSEMS or PS placement. We believe that none of the patients in our study developed pancreatitis because intraductal placement does not obstruct the pancreatic duct. Intraductal placement is preferable to prevent retrograde cholangitis.

**FIGURE 7:**
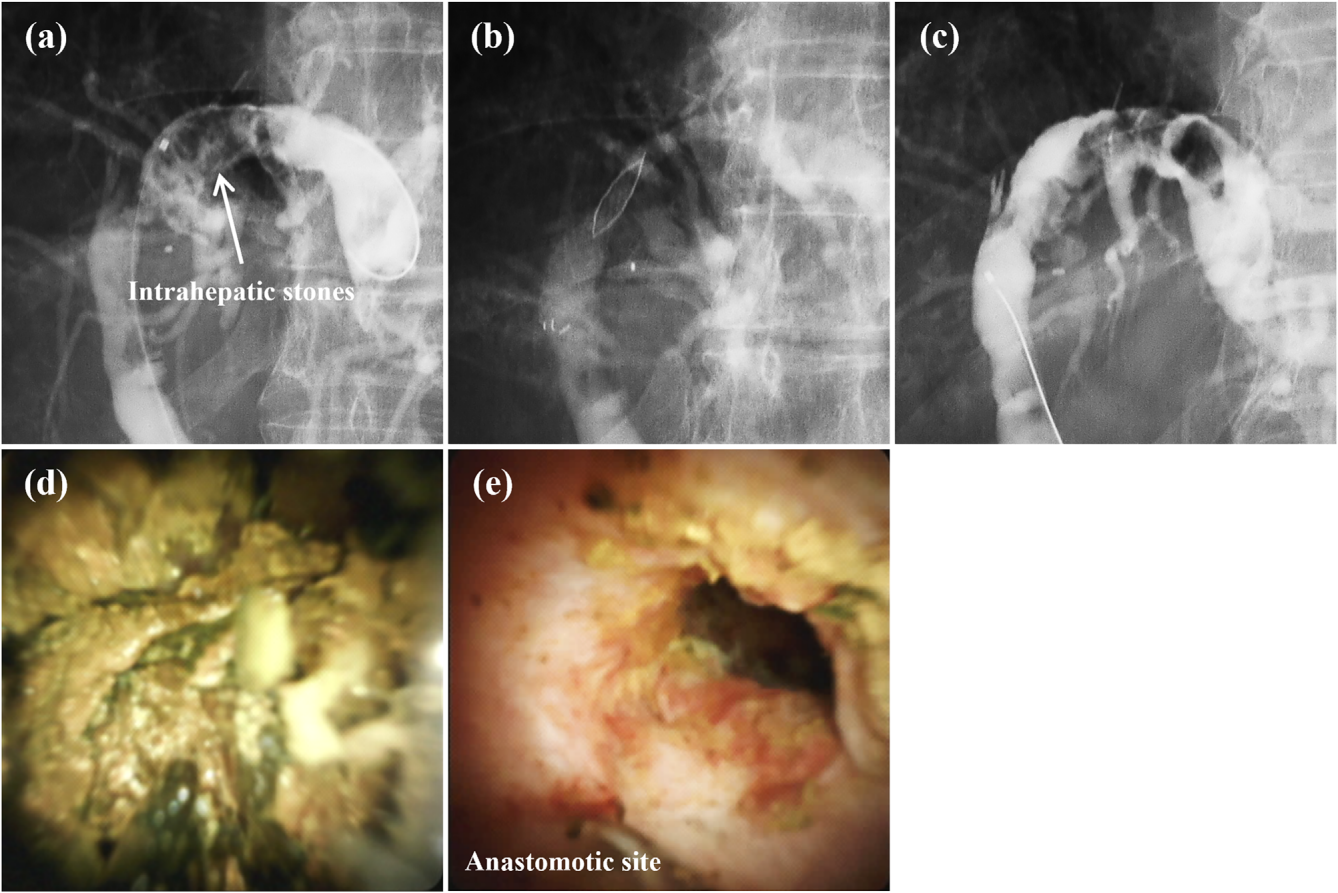
This patient was a 70‐year‐old male with an anastomotic biliary stricture, which developed after left‐lobe living donor liver transplantation, and large intrahepatic stones (arrow). (a) The intrahepatic stones were seen on the hepatic side of the biliary stricture 7 years after liver transplantation and cholangitis began to recur. (b) An endoscopic retrograde cholangiopancreatography was performed and a fully covered self‐expandable metallic stent (FCSEMS) was placed above the papilla. (c) The FCSEMS was removed at 16 weeks, as scheduled. The anastomotic biliary stricture was completely resolved, and the SpyGlass DSTM could be inserted easily. (d) Intrahepatic stones were crushed using electrohydraulic lithotripsy and removed with a balloon or basket catheter. (e) Complete stone clearance was achieved with a single treatment. Recurrence of the bile duct stones and biliary stricture has not been observed as of 18 months after the procedure.

The incidence of bile duct stones after LT is 10%^35^ and more than half of these patients experience biliary strictures. An FCSEMS is placed with a balloon or basket after the removal of common bile duct stones, however, since intrahepatic stones are located proximally to the stricture, they are difficult to remove. Intrahepatic stones were observed in three patients. In two of these cases, the stones were large and difficult to remove with a balloon or basket catheter, so we crushed and removed them with a peroral cholangioscopy using a SpyGlass DS and electrohydraulic lithotripsy (Figure 7). We believe the SpyGlass DS would not have been able to break through the stricture if the FCSEMS had not been placed. The median anastomotic diameter after FCSEMS removal was 6.3 mm (range, 3.5–7.1 mm). As the maximum diameter of the SpyGlass DS is 3.6 mm, it passed beyond the anastomotic site without resistance. We were able to insert a cholangioscopy without performing EST because the papilla was loosened by multiple ERCPs. Therefore, the FCSEMS is advantageous because it allows simultaneous stricture resolution and bile duct stone removal.

In conclusion, FCSEMSs were placed in nine patients with ABS for whom plastic stents after LDLT failed, and 100% technical and clinical success rates were achieved. Stricture recurrence was not observed in eight of the patients. Notably, FCSEMSs can be placed repeatedly, even for cases of recurrence. This study makes a novel contribution to the literature by setting the FCSEMS placement period to 16 weeks for ABS after LDLT. Our results suggest that FCSEMSs are useful for treating refractory ABSs after LDLT. Additionally, FCSEMS placement for initial treatment may be considered, although more studies are required with larger sample sizes.

## CONFLICT OF INTEREST STATEMENT

None.

## References

[1] Jain A , Reyes J , Kashyap R *et al.* Long‐term survival after liver transplantation in 4,000 consecutive patients at a single center. Ann Surg 2000; 232: 490–500. 10998647 10.1097/00000658-200010000-00004 PMC1421181

[2] Mazzaferro V , Bhoori S , Sposito C *et al.* Milan criteria in liver transplantation for hepatocellular carcinoma: An evidence‐based analysis of 15 years of experience. Liver Transpl 2011; 17: S44–57. 21695773 10.1002/lt.22365

[3] Akamatsu N , Sugawara Y , Hashimoto D . Biliary reconstruction, its complications and management of biliary complications after adult liver transplantation: A systematic review of the incidence, risk factors and outcome. Transpl Int 2011; 24: 379–92. 21143651 10.1111/j.1432-2277.2010.01202.x

[4] Thethy S , Thomson B , Pleass H *et al.* Management of biliary tract complications after orthotopic liver transplantation. Clin Transplant 2004; 18: 647–53. 15516238 10.1111/j.1399-0012.2004.00254.x

[5] Hampe T , Dogan A , Encke J *et al.* Biliary complications after liver transplantation. Clin Transplant 2006; 20: 93–6. 17100708 10.1111/j.1399-0012.2006.00607.x

[6] Kochhar G , Parungao JM , Hanouneh IA , Parsi MA . Biliary complications following liver transplantation. World J Gastroenterol 2013; 19: 2841–6. 23704818 10.3748/wjg.v19.i19.2841 PMC3660810

[7] Verdonk RC , Buis CI , Porte RJ *et al.* Anastomotic biliary strictures after liver transplantation: Causes and consequences. Liver Transpl 2006; 12: 726–35. 16628689 10.1002/lt.20714

[8] Sharma S , Gurakar A , Jabbour N . Biliary strictures following liver transplantation: Past, present and preventive strategies. Liver Transpl 2008; 14: 759–69. 18508368 10.1002/lt.21509

[9] Chang JH , Lee IS , Choi JY *et al.* Biliary stricture after adult right‐lobe living‐donor liver transplantation with duct‐to‐duct anastomosis: Long‐term outcome and its related factors after endoscopic treatment. Gut Liver 2010; 4: 226–33. 20559526 10.5009/gnl.2010.4.2.226 PMC2886928

[10] Zimmerman MA , Baker T , Goodrich NP *et al.* Development, management, and resolution of biliary complications after living and deceased donor liver transplantation: A report from the adult‐to‐adult living donor liver transplantation cohort study consortium. Liver Transpl 2013; 19: 259–67. 23495079 10.1002/lt.23595 PMC3602918

[11] Hsieh TH , Mekeel KL , Crowell MD *et al.* Endoscopic treatment of anastomotic biliary strictures after living donor liver transplantation: Outcomes after maximal stent therapy. Gastrointest Endosc 2013; 77: 47–54. 23062758 10.1016/j.gie.2012.08.034

[12] Holt AP , Thorburn D , Mirza D , Gunson B , Wong T , Haydon G . A prospective study of standardized nonsurgical therapy in the management of biliary anastomotic strictures complicating liver transplantation. Transplantation 2007; 84: 857–63. 17984838 10.1097/01.tp.0000282805.33658.ce

[13] Kim TH , Lee SK , Han JH *et al.* The role of endoscopic retrograde cholangiography for biliary stricture after adult living donor liver transplantation: Technical aspect and outcome. Scand J Gastroenterol 2011; 46: 188–96. 20955089 10.3109/00365521.2010.522722

[14] Albert JG , Filmann N , Elsner J *et al.* Long‐term follow‐up of endoscopic therapy for stenosis of the biliobiliary anastomosis associated with orthotopic liver transplantation. Liver Transpl 2013; 19: 586–93. 23585381 10.1002/lt.23643

[15] Kurita A , Kodama Y , Minami R *et al.* Endoscopic stent placement above the intact sphincter of Oddi for biliary strictures after living donor liver transplantation. J Gastroenterol 2013; 48: 1097–104. 23325164 10.1007/s00535-012-0705-x

[16] Tsujino T , Isayama H , Kogure H , Sato T , Nakai Y , Koike K . Endoscopic management of biliary strictures after living donor liver transplantation. Clin J Gastroenterol 2017; 10: 297–311. 28600688 10.1007/s12328-017-0754-z

[17] Devière J , Reddy DN , Püspök A *et al.* Successful management of benign biliary strictures with fully covered self‐expanding metal stents. Gastroenterology 2014; 147: 385–95. 24801350 10.1053/j.gastro.2014.04.043

[18] Aepli P , St John A , Gupta S *et al.* Success and complications of an intra‐ductal fully covered self‐expanding metal stent (ID‐FCSEMS) to treat anastomotic biliary strictures (AS) after orthotopic liver transplantation (OLT). Surg Endosc 2017; 31: 1558–63. 27572066 10.1007/s00464-016-5138-9

[19] Tal AO , Finkelmeier F , Filmann N *et al.* Multiple plastic stents versus covered metal stent for treatment of anastomotic biliary strictures after liver transplantation: A prospective, randomized, multicenter trial. Gastrointest Endosc 2017; 86: 1038–45. 28302527 10.1016/j.gie.2017.03.009

[20] Moon JH , Choi HJ , Koo HC *et al.* Feasibility of placing a modified fully covered self‐expandable metal stent above the papilla to minimize stent‐induced bile duct injury in patients with refractory benign biliary strictures (with videos). Gastrointest Endosc 2012; 75: 1080–5. 22401821 10.1016/j.gie.2012.01.016

[21] Sato T , Kogure H , Nakai Y *et al.* A prospective study of fully covered metal stents for different types of refractory benign biliary strictures. Endoscopy 2020; 52: 368–76. 32092770 10.1055/a-1111-8666

[22] Wan P , Yu X , Xia Q . Operative outcomes of adult living donor liver transplantation and deceased donor liver transplantation: A systematic review and meta‐analysis. Liver Transpl 2014; 20: 425–36. 24478109 10.1002/lt.23836

[23] Facciorusso A , Rosca EC , Ashimi A *et al.* Management of anastomotic biliary stricture after liver transplantation: Metal versus plastic stent. Ann Gastroenterol 2018; 31: 728–34. 30386124 10.20524/aog.2018.0297 PMC6191877

[24] Sato T , Kogure H , Nakai Y *et al.* Long‐term outcomes of endoscopic treatment for duct‐to‐duct anastomotic strictures after living donor liver transplantation. Liver Int 2019; 39: 1954–63. 31436017 10.1111/liv.14219

[25] Chaput U , Scatton O , Bichard P *et al.* Temporary placement of partially covered self‐expandable metal stents for anastomotic biliary strictures after liver transplantation: A prospective, multicenter study. Gastrointest Endosc 2010; 72: 1167–74. 20970790 10.1016/j.gie.2010.08.016

[26] Yoo JJ , Lee JK , Moon JH *et al.* Intraductal placement of non‐flared fully covered metallic stent for refractory anastomotic biliary strictures after living donor liver transplantation: Long‐term results of prospective multicenter trial. J Gastroenterol Hepatol 2020; 35: 492–8. 31418477 10.1111/jgh.14831

[27] Landi F , de'Angelis N , Sepulveda A *et al.* Endoscopic treatment of anastomotic biliary stricture after adult deceased donor liver transplantation with multiple plastic stents versus self‐expandable metal stents: A systematic review and meta‐analysis. Transpl Int 2018; 31: 131–51. 29090502 10.1111/tri.13089

[28] Jang S , Stevens T , Lopez R *et al.* Self‐expandable metallic stent is more cost efficient than plastic stent in treating anastomotic biliary stricture. Dig Dis Sci 2020; 65: 600–8. 31104197 10.1007/s10620-019-05665-9

[29] Matsumoto K , Kato H , Fujii M *et al.* Efficacy of intraductal placement of nonflared fully‐covered metal stent for refractory perihilar benign biliary strictures: A multicenter prospective study with long‐term observation. J Hepatobiliary Pancreat Sci 2022; 29: 1300–7. 35657019 10.1002/jhbp.1188

[30] Jang S , Parsi MA , Lopez R , Bhatt A , Vargo JJ . Efficacy and optimal duration of metallic stent in the management of refractory anastomotic stricture after liver transplantation. Clin Gastroenterol Hepatol 2017; 15: 1776–81. 28624651 10.1016/j.cgh.2017.06.015

[31] Hu B , Gao DJ , Yu FH , Wang TT , Pan YM , Yang XM . Endoscopic stenting for post‐transplant biliary stricture: Usefulness of a novel removable covered metal stent. J Hepatobiliary Pancreat Sci 2011; 18: 640–5. 21643818 10.1007/s00534-011-0408-3

[32] Moon JH , Choi HJ , Koo HC *et al.* Feasibility of placing a modified fully covered self‐expandable metal stent above the papilla to minimize stent‐induced bile duct injury in patients with refractory benign biliary strictures (with videos). Gastrointest Endosc 2012; 75: 1080–5. 22401821 10.1016/j.gie.2012.01.016

[33] Jiménez‐Pérez M , Melgar Simón JM , Durán Campos A , González Grande R , Rodrigo López JM , Manteca González R . Endoscopic management of post‐liver transplantation biliary strictures with the use of fully covered metallic stents. Transplant Proc 2016; 48: 2510–4. 27742337 10.1016/j.transproceed.2016.09.008

[34] Martins FP , De Paulo GA , Contini MLC , Ferrari AP . Metal versus plastic stents for anastomotic biliary strictures after liver transplantation: A randomized controlled trial. Gastrointest Endosc 2018; 87: 131.e1–.e13. 10.1016/j.gie.2017.04.013 28455159

[35] Senoo T , Ichikawa T , Taura N *et al.* Incidence of and risk factors for bile duct stones after living donor liver transplantation: An analysis of 100 patients. Hepatol Res 2015; 45: 969–75. 25331775 10.1111/hepr.12438

